# Protecting our future generation: study protocol for a randomized controlled trial evaluating a sexual health self-care intervention with Native American youth and young adults

**DOI:** 10.1186/s12889-019-7956-x

**Published:** 2019-12-02

**Authors:** Tingey Lauren, Sutcliffe Catherine, Chambers Rachel, Patel Hima, Lee Angelita, Lee Shauntel, Melgar Laura, Slimp Anna, Rompalo Anne, Craig Mariddie, Gaydos Charlotte

**Affiliations:** 1 Johns Hopkins Center for American Indian Health, Johns Hopkins University, Bloomberg School of Public Health, 415 N. Washington St., Baltimore, MD 21231 USA; 2 Johns Hopkins Center for American Indian Health, Johns Hopkins University, Bloomberg School of Public Health, 308 Kuper St., Whiteriver, AZ 85941 USA; 3 Johns Hopkins Center for the Development of Point Of Care Tests for Sexually Transmitted Diseases, Johns Hopkins University, Bloomberg School of Public Health, 855 N. Wolfe St., Baltimore, MD 21205 USA

**Keywords:** Sexually transmitted infection, Native American, Youth, Young adults, Self-care, Screening, Risk and protective factors

## Abstract

**Background:**

Disparities in sexually transmitted infections (STI) are an urgent problem among Native American youth and young adults which are not fully explained by different sexual or related behaviors. These sexual health disparities are more likely attributed to social environments and structural determinants such as a shortage of sexual healthcare providers, lower socioeconomic status, and access barriers to STI screening and treatment, including geographic isolation and confidentiality concerns. Innovative, non-clinic based alternatives to promote STI screening and treatment are essential for alleviating these disparities. Self-care, or the care taken by individuals towards their own health and well-being may be such a strategy. This study will assess the efficacy of a self-care intervention, called Protecting Our Future Generation, for increasing uptake of STI screening and impacting sexual risk and protective behaviors among Native American youth and young adults living in a reservation-based community in the Southwestern United States.

**Methods:**

The proposed study is a randomized controlled trial to test the efficacy of a self-care intervention compared to a control condition. Participants will be Native Americans ages 14–26 years old who have had vaginal or anal sex at least once in their lifetime. Participants will be randomized to the intervention which includes: 1) a sexual health self-assessment with embedded clinical prediction tool predicting STI positivity, and 2) personalized messaging with key steps to lower risk for STIs, or the control condition which includes: 1) a self-assessment about water, soda and sugar sweetened beverage consumption, and 2) personalized messaging to meet recommended daily intake. All participants will be offered a self-administered STI test. Participants will complete assessments at baseline, 3- and 6-months follow-up. The primary outcome measure is completion of STI screening.

**Discussion:**

Protecting Our Future Generation is among the first self-care interventions uniquely focused on sexual health among a Native American population, who endure significant sexual health disparities and are under-represented in research. If efficacious, the intervention will be a model of sexual health self-care for Native American youth and young adults adaptable for use in healthcare and community-based settings.

**Trial registration:**

Clinical Trials: http://clinicaltrials.gov; NCT03895320; Registered 03/28/2019.

## Background

Disparities in sexually transmitted infections (STI) are an urgent problem among Native American (Native) youth and young adults. Nationwide, in 2017, the Chlamydia rate among Native Americans was 47.7% higher than the general population (781.2/100,000 vs. 528.8/1000) and rates of Chlamydia rose 3.7% among Natives between 2013 and 2017 [1]. Further, Natives had the second highest Gonorrhea rate among all races/ethnicities in 2017 [1].

Native young adults and adolescents suffer from high rates of Gonorrhea, with a rate of 561.9/100,000 for those between ages 15–19, and 926.6/100,000 for those between ages 20–24; these rates are 4.2 and 3.6 times the rate among Whites, respectively [1]. In 2013, in Arizona, where this study will take place, Native American females ages 20–24 endured the highest rates of Chlamydia among any demographic in Arizona, at 7767/100,000, which is 1.5 times the rate for Native females of the same age nationwide (5310/100,000) [2]. Additionally in Arizona, from 2012 to 2013 the greatest increase in Gonorrhea rates were among Native Americans (23%) [2].

Research attempting to explain this gap in STIs indicates a higher prevalence of STIs among Native youth and young adults is not fully explained by different sexual or substance use behaviors [3]. These disparities in sexual health by ethnicity may be better attributed to social environments and structural determinants such as a shortage of sexual healthcare providers, socioeconomic status, and access barriers to healthcare including confidentiality concerns, and geographic isolation [4–10]. Further, because many STIs can be treated free of charge within the Indian Health Service (IHS), differential access to screening and treatment may explain these disparities [3]. Data from IHS corroborate inequalities in access to care, namely underutilization of services by higher risk groups and a lack of universal promotion of sexual health care by providers [4, 11].

Innovative, non-clinic based alternatives to traditional sexual health screening and treatment are essential for alleviating these sexual health disparities. Promotion of self-care, or the care taken by individuals towards their own health and well-being may be such a strategy; it is endorsed for improving sexual health outcomes by increasing access to care and individual satisfaction with care through client-driven, confidential options [4, 12–16]. Improving sexual health though the promotion of self-care is increasingly a focus of preventing sexual health disparities due to: 1) increased availability of new technologies, 2) the ease of STI screening using non-invasive samples, and 3) the lack of capacity for routine sexual health care service provision common in many reservation-based and other rural communities [13].

Approaches that focus on self-care may also address budgetary limitations at IHS by maximizing case finding through selective screening of individuals at highest risk, aiding in risk communication with providers, and stimulating health-seeking behavior [4, 12–16]. Self-care approaches can also include strategies for identifying those at increased risk for STIs, and have potential for reducing onward transmission and preventing new cases of disease [15, 16]. Risk prediction or clinical prediction rules are tools that provide estimates of absolute risk based on a combination of several individual characteristics and are a nuanced approach to sexual health self-care [14–16]. Specifically, a brief self-assessment including a clinical prediction rule and corresponding personalized messaging may: 1) help an individual gain knowledge of their own risks; 2) stimulate health-care seeking behavior such as STI screening and treatment; and 3) grow patient efficacy for taking charge of their sexual health.

Many sexual health care interventions are developed from risk or deficits-based perspectives [17–21]. An alternative, protective factors-based approach, such as that rooted in Asset Theory, would instead focus on the strengths, assets and resources of individuals and their connections within their community [22]. With regard to youth and young adults, a focus on internal knowledge, attitudes, and positive behavioral choices, in addition to connection with pro-social peers and adults, communication with parents and other family members, and involvement in community activities may bear greater import and impact on sexual health outcomes of interest [17, 22, 23]. A focus on the aforementioned assets and resources may drive positive changes in sexual health by motivating individual attitudes to avoid negative health outcomes and providing strategies to utilize available health care [17, 22–25]. Such an approach is compatible with Native views of health and well-being which focus on strengths and a balance between physical, social, emotional and environmental health [18–21, 26]. Past research conducted with youth, including Native American youth, supports this idea and shows a predictive relationship between youth’s strengths and resources and the protective sexual health outcomes of abstinence, delayed sexual initiation, and use of birth control [22, 23].

### The current study

A self-care intervention including self-assessment and personalized messaging, grounded in Asset Theory, may motivate the protective sexual health behavior of STI screening. Specifically, risk prediction tools delivered through a self-assessment and corresponding personalized messaging have the potential to be powerful decision aids and should be evaluated using experimental study designs. This study will assess the efficacy of a self-care intervention, called Protecting Our Future Generation (POFG), for increasing uptake of STI screening and impacting sexual risk and protective behaviors, psychosocial assets, and resources. This paper describes the intervention Protecting Our Future Generation and protocol for its implementation and evaluation.

### Study aims

This study will be conducted in the southwestern U.S. with Native American youth and young adults (ages 14–26) residing in a rural, reservation-based community. We will conduct a two-arm randomized controlled trial to evaluate the POFG program for impacts on STI screening uptake, sexual health risk behaviors and protective sexual health practices. The primary research questions include: 1) Does POFG increase STI screening uptake; 2) Does POFG increase condom use at last sex; and 3) Does POFG decrease number of new sexual partners. Secondary research questions include exploring what participant variables impact the primary outcome of STI screening uptake, including: 1) sexual health knowledge, efficacy and intentions; 2) substance use; 3) psychosocial assets and resources; and 4) experiencing past symptoms of a STI.

## Methods/design

### Study overview and hypotheses

**Fig. 1:**
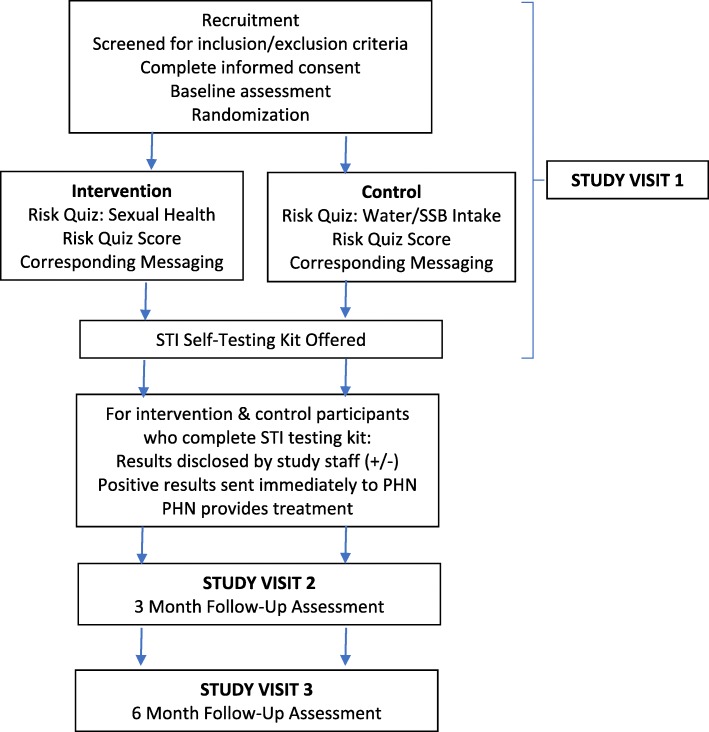
Describes the participant flow through the various activities of the study

We will conduct a two-arm randomized controlled trial (RCT) to test the efficacy of the POFG self-care intervention for impacting participant uptake of STI screening, in comparison to a control group. POFG includes a brief self-assessment to predict absolute STI risk and corresponding personalized messaging. We hypothesize participants who receive POFG will have a higher rate of STI screening completion at 3-months follow-up, compared with participants who receive the control program. We will measure STI screening uptake at 6-months follow-up to see if any increases were maintained over time. We hypothesize that POFG will increase protective behaviors among participants by: i) helping youth and young adults gain knowledge of their own sexual health risks, assets and resources; ii) motivating protection of those assets and resources; and iii) encouraging good health practices and making responsible choices. We hypothesize the effects of POFG on STI screening uptake will be mediated by participant’s substance use and past STI infection. See Fig. 1 for an illustration of the flow of participants through the study.

The study will be conducted through a partnership between Johns Hopkins Center for American Indian Health (JHU) and a tribal community residing on a reservation in rural Arizona. The trial design and study protocol were reviewed and approved by the Tribe’s governing Tribal Council and Health Advisory Board as well as the JHU and IHS research review boards. This manuscript was reviewed and approved by the Tribal Council and Health Advisory Board. Any serious adverse event reported or detected during the trial will be documented and reported to the relevant research review boards within 24 h. There is no Data and Safety Monitoring Board for this trial. (Note: a completed SPIRIT checklist was submitted along with the manuscript to the journal).

### Participant recruitment and informed consent

To participate in the study participants must meet the following criteria: Native American ethnicity, 14–26 years of age, member of the participating tribal community, written informed consent, had vaginal or anal sex at least once in their lifetime, and be in possession of their own cell phone. We will utilize a non-probability sampling frame that includes posting flyers in public and conducting in-person recruitment at community gathering locations. Study staff will screen potential participants for eligibility criteria. If eligible and interested, individuals will complete informed consent. Note: The relevant Institutional Review Boards (IRB) approved for minors ages 14–17 to consent to study participation as adults in lieu of requiring parental permission. This decision is based on state law allowing individuals ages 14 and older to consent to STI screening without parental permission. Thus, the IRBs believed requiring parental permission could potentially act as a barrier to study participation and preclude access to the benefit of STI screening. The first participant was recruited on April 1, 2019.

### Participant randomization and study sample size

This study is at RCT. The randomization sequence is computer generated and used to randomize individuals to one of two study groups: intervention or control. A stratified randomization technique will be used to ensure equivalent 1:1 allocation of study conditions across three gender groups (male/trans male; female/trans female and other, which includes those who identify as two-spirited, bi-gendered, crossdresser, genderqueer, or for whom none of these options apply), and three age groups (i.e. 14–17, 18–21, and 22–26). Within each age/gender strata, participants will be randomized in blocks of 4 to ensure equal allocation to intervention and control groups. Participants, but not study staff or investigators, will be blinded to study group participation.

The primary outcome is completion of self-administered STI screening between the baseline and 3-month study visit. Based on prior research, we estimate that 20–25% of the control group will complete STI screening [12]. We anticipate POFG will increase STI screening by at least 50% (i.e. relative risk of 1.5 comparing the proportion completing STI screening in the intervention group to the control group). Given these assumptions, retention rates from past RCTs conducted by the study team within the target population, a two-sided alpha of 0.05 and 80% power, we will enroll a total of 450 participants (225 in each arm) to sufficiently power the trial.

### Intervention

Protecting Our Future Generation (POFG) is a self-care intervention rooted in Asset Theory [17–25]. POFG provides participants with an opportunity to assess their own sexual risk and protective behaviors, understand their personal risk for current or future STI infection, receive tailored strategies and recommendations to engage in protective sexual health practices, and motivate completion of non-clinic based STI screening. The self-assessment includes questions pertaining to sexual health risk and protective behaviors and a valid clinical prediction tool established to predict STI positivity [16]. The personalized messaging describes key steps the person can take to lower their risk for STIs. The quiz with resulting score and personalized messaging will take approximately ten minutes to complete and be delivered via tablet at the first study visit. POFG participants will be offered a self-administered, non-clinic based STI testing kit (for *Neisseria gonorrhea*, *Chlamydia trachomatis* and *Trichomonas vaginalis*) after receiving their personalized messages.

### Control condition

The control program includes: a) a brief self-assessment (also called ‘quiz’), b) quiz score, and c) personalized messaging. For the control condition, the quiz, score and messaging will pertain to consumption of water, soda and sugar sweetened beverages. There will not be a comparable clinical prediction tool in the quiz for the control condition. However, based on the answers given on the control quiz, key steps the control participant can take to meet the recommended daily intake of water and sugar sweetened beverages will be delivered. The control quiz with resulting score and personalized messaging will take approximately ten minutes to complete and will be delivered via tablet at the first study visit. Control participants will also be offered a self-administered, non-clinic based STI testing kit (for *Neisseria gonorrhea*, *Chlamydia trachomatis* and *Trichomonas vaginalis*) after receiving their personalized messages. The content and structure of the control condition was recommended by the local study team from the participating tribal community and endorsed as a beneficial comparison to the intervention.

The quality of both programs (intervention and control) will be ensured through self-administration via tablet. Thus, delivery of each quiz and corresponding messaging is standardized across participants in each group.

### STI treatment

For participants who opt to take the STI testing kit (intervention or control) they can: 1) complete it and return it to a study staff immediately, 2) take the test with them, complete it at a later time and drop it off at the designated study office, or 3) complete it at a later time and coordinate with a study staff to have it picked up at a location they choose.

All participants (intervention and control) who test positive for a STI will receive an assisted referral to a Public Health Nurse at IHS with whom we have partnered on this study to ensure all participants who test positive for one or more STIs receive timely and high-quality treatment.

### Measures

The following assessments will be completed by participants: the standardized *Youth Health Risk Behavior Inventory* which has been previously adapted and piloted with Native American youth and young adults yielding Cronbach’s alphas ranging from 0.74–0.93; and the standardized *Youth Asset Survey*, with established reliability and validity among a random sample of 1350 youth (10% of which were Native; Cronbach’s alpha > 0.61) [17, 27, 28]. These two self-report surveys will collect information on participant demographics, sexual risk and protective behaviors, including STI screening uptake, as well as psychosocial assets and resources. All survey items were piloted with ten youth and young adults from the participating community (3 males and 7 females) ranging in age from 14 to 24 years; edits were made as necessary. See Table 1 for a complete description of measures.

**Table 1:** Protecting Our Future Program Evaluation Measures

Measures	Description of Measure
Youth Health Risk Behavior Inventory	Assesses sexual behavior and related risk factors through close-ended questions that asks about STI and sexual behavior history, knowledge, intentions and attitudes. Adapted from Stanton and colleagues [27].
Youth Asset Survey	Assesses youth assets (knowledge, attitudes, future aspirations, use of time, health practices and responsible choices (role models, communication with parents and adults, community involvement and access to health care). Adapted from Oman and colleagues [17].

### Study visits and data collection

Data will be collected at baseline, 3 months, and 6 months post-intervention completion. At each study visit, participants will complete the aforementioned surveys. Data will be collected via tablets using REDcap (Research Electronic Data Capture) electronic data capture tools hosted at JHU [29]. REDCap is a secure, web-based application designed to support data capture for research studies, providing 1) an intuitive interface for validated data entry; 2) audit trails for tracking data manipulation and export procedures; 3) automated export procedures for seamless data downloads to common statistical packages; and 4) procedures for importing data from external sources. Participants will receive $30, $30 and $45 gift cards after completion of the baseline, 3- and 6-month post-intervention evaluations, respectively.

### Outcomes

#### Primary outcome

Completion of STI screening between the baseline and 3-month study visit is the primary outcome for this study.

#### Secondary outcomes

Secondary outcomes include: 1) number of times in past 3 months had sex without a condom, 2) number of times in past 3 months had sex without birth control, 3) number of partners in past 3 months, 4) number of new partners in past 3 months, 5) current symptoms of STI, 6) alcohol and drug use in past 3 months, 7) alcohol and drug use before sex in past 3 months, 8) condom use self-efficacy, 9) condom use intention, 10) attitudes towards condom use, 11) belief condoms prevent STI/pregnancy,12) birth control intention, 13) STI screening intention, 14) sexual health knowledge, 15) connectedness to school, 16) use of time in sports/recreational/religious activities, 17) family communication, 18) peer role model/influence, 19) healthy peer norms, and 20) aspirations for the future.

### Statistical analysis

Study hypotheses will be tested using an “intent to treat” analysis. Baseline characteristics will be compared between groups using chi-square tests for binary or categorical outcomes, and Wilcoxon rank-sum tests for continuous outcomes. For the primary outcome of STI screening uptake, the proportion of participants completing screening (i.e. returning a test to study staff) by the 3-month study visit will be compared across groups using a chi-square test or log-binomial regression if participant characteristics are found to vary at baseline between groups. For all analyses, participants who drop-out during the study and don’t provide at least 3-month follow-up data will be removed from the analysis. If at 3- or 6-months follow-up the POFG intervention shows efficacy for increasing STI screening uptake, we will conduct mediation and moderation analyses to explore what factors may be impacting the efficacy of POFG to change STI screening behavior.

## Discussion

This study protocol has many strengths. First, Protecting Our Future Generation is among the first self-care interventions uniquely focused on sexual health among Native American youth and young adults who endure significant sexual health disparities and are under-represented in sexual health research [15]. Second, this study draws on an unconventional and nuanced approach to sexual health self-care through the use of self-assessments combined with personalized messaging, and self-administered non-clinic based STI screening. These approaches have the potential for widespread uptake within communities served by Indian Health Service, and provide complementary alternatives to provider-driven, clinic-based care [4, 11, 13, 16]. Third, the self-assessment will include a risk prediction/clinical prediction rule to assist with identifying those at greatest risk for STIs, which is urgently needed in this and other settings where STI rates are climbing. Although clinical prediction rules have the capability to minimize resource use and slow the spread of infection, they are infrequently developed and uncommonly used in sexual health care; our study has potential to convert clinical prediction rules into a client-driven tool for improving uptake of sexual health care [15].

Fourth, this research is rooted in Asset Theory, an under-utilized framework for understanding sexual health risk and protective behaviors, and a model which may be particularly suited to Native and other indigenous communities [25]. A focus on assets and resources acknowledges the social/environmental determinants of sexual health and is more congruent with Native American and other indigenous belief systems, as well as a necessary move away from a focus on static, individual traits [26]. Research shows assets and resources can: 1) create future orientation by shaping opportunity structures, 2) stimulate maintenance of existing assets and resources by motivating prudent and protective behavior, 3) provide a foundation for risk-taking, and 4) increase personal efficacy [24, 25]. Despite research showing a predictive relationship between number of assets and resources and protective sexual health practices among youth, our study would be the first to do this within a Native American context [23, 26].

A limitation of this study is that data are collected via self-report and subject to response bias. The study uses tablets and REDcap technology so that surveys can be self-administered in an attempt to minimize this bias. Another limitation is that the intervention involves different components and we won’t necessarily be able to tease apart the impact of each individually (i.e. quiz with clinical risk prediction tool vs. messaging).

If efficacious, POFG may be a model of sexual health self-care for Native American youth and young adults adaptable for use in diverse settings. The use of self-administered sample collection for STI screening, which our study team has proven acceptable and feasible with the participating tribal community, further enhances the ability of Native youth and young adults to take their sexual health into their own hands [28]. The design of POFG, which is tablet-based, brief and confidential, allows for ready implementation through various systems, including: schools, IHS’s Public Health Nursing department, and Tribal Community Health Representatives and Division of Health. To the study team’s knowledge, this is the first rigorous evaluation of a sexual health self-care intervention that incorporates self-administered STI screening for Native American youth and young adults.

## Data Availability

Not applicable.

## Abbreviations

IRB: Institutional Review Board
JHU: Johns Hopkins University/Johns Hopkins Center for American Indian Health
POFG: Protecting Our Future Generation
RCT: Randomized Controlled Trial
REDCap: Research Electronic Data Capture
STI: Sexually Transmitted Infection
